# Safety of opicapone in the elderly and very elderly population: an analysis of reported events in a global safety database

**DOI:** 10.1016/j.prdoa.2026.100500

**Published:** 2026-09-09

**Authors:** Taku Hatano, Peter A. LeWitt, Tetsuya Maeda, Javier Pagonabarraga Mora, Bruno F. Dias, Helena C. Brigas, Diogo Lopes, Helena Gama, Joerg Holenz

**Affiliations:** a Department of Neurology, Juntendo University Faculty of Medicine, Tokyo, Japan; b Wayne State University School of Medicine, Departments of Neurology and Pharmacology, Sastry Foundation Endowed Chair in Parkinson's Disease Research, Detroit, MI, USA; c Division of Neurology and Gerontology, Department of Internal Medicine, School of Medicine, Iwate Medical University, Iwate, Japan; d Movement Disorders Unit, Neurology, Hospital Santa Creu i Sant Pau, Universitat Autònoma de Barcelona (Department of Medicine), Barcelona, CIBERNED, Spain; e BIAL - Portela & C^a^, S.A., Coronado, Portugal

**Keywords:** COMT-inhibitor, Levodopa, Opicapone, Parkinson's disease, Safety

## Abstract

**Introduction:**

Older people with Parkinson's disease (PD) are under-represented in clinical trials despite increasing disease burden and complexity. Although opicapone is approved as an adjunct to levodopa, data in elderly populations are limited. This study assessed its real-world safety in the elderly using post-marketing pharmacovigilance data.

**Methods:**

This retrospective analysis used the global opicapone safety database (data-lock: 31 Jan 2026). Valid case safety reports with opicapone as the suspected product were included and classified as elderly (≥65–84 years) or very elderly (≥85 years). Adverse events were analyzed descriptively by system organ class, preferred term, and seriousness. Non-standard use and treatment actions were also assessed.

**Results:**

Among 2914 analyzed cases (2558 elderly; 356 very elderly), 7195 adverse events were reported. Most reported events were non-serious (81.4% of events in elderly cases; 73.5% in very elderly cases). Dyskinesia was the most frequently reported event in both groups (7.1%–7.7% of events), followed by hallucination and visual hallucination (all <5% of events). Serious events accounted for a greater proportion of reported events in the very elderly versus elderly group (26.5% vs 18.6%), although no individual preferred term exceeded 5%. Non-standard use accounted for 6.0% and 3.5% of reported events in the very elderly and elderly groups, respectively, and included altered administration and off-label indications. Treatment was discontinued in approximately half of cases in both groups.

**Conclusion:**

Reported adverse event patterns were generally consistent with the established safety profile of opicapone across both age groups. No unexpected reporting patterns were observed; however, findings should be interpreted within the limitations of spontaneous pharmacovigilance data.

## Highlights

• Global pharmacovigilance data showed broadly similar adverse event patterns in elderly and very elderly PwP.
• Most adverse events were non-serious, and consistent with established dopaminergic effects.
• No unexpected adverse events or reporting patterns were observed in this dataset.
• Serious AEs were slightly more frequent in very elderly PwP but individual events remained infrequent (<5%).
• Findings support continued, monitored use of opicapone in older populations, despite real-world complexity.

## 1 Introduction

With the ageing global population, the prevalence of Parkinson's disease (PD) is projected to grow rapidly [1]. Modelling based on the Global Burden of Disease Study predicts that individuals aged ≥80 years will experience the greatest increase in PD burden, with the number of cases estimated to rise by 196% between 2021 and 2050 [1]. This demographic shift is accompanied by greater clinical complexity, including multimorbidity, frailty, cognitive impairment, and polypharmacy, all of which can complicate pharmacological management and increase vulnerability to adverse drug events. Yet, older and very elderly people with Parkinson's (PwP) remain under-represented in randomized controlled trials (RCTs), limiting age-specific safety data.

Levodopa remains the cornerstone of treatment for all PwP. However, ageing-related physiological changes may increase vulnerability to functional deterioration and treatment complications associated with levodopa [2]. Its variable pharmacokinetic profile contributes to inconsistent symptom control, making careful optimization of dosing regimens essential, particularly to address OFF episodes in elderly and very elderly populations.

Opicapone, a once-daily, peripherally acting catechol-*O*-methyltransferase (COMT) inhibitor, is approved as an adjunct to levodopa in fluctuating PwP. Subgroup analyses from pivotal trials have demonstrated clinically meaningful reductions in OFF-time in older adults aged ≥70 years, with efficacy consistent with that observed in the overall study populations [3], [4]. While opicapone clinical trials have included PwP up to 83 years of age (with a comparable overall incidence of adverse events [AEs] in those aged ≥70 years versus placebo [4]), data in very elderly PwP remain sparse. In this context, post-marketing pharmacovigilance data offers an important complement to RCTs by capturing the broader and more heterogeneous patient population who regularly use opicapone. This includes PwP of advanced age, with multiple comorbidities and complex treatment regimens. It also allows the identification of safety patterns that may not be readily observable during the clinical development process.

The present analysis of the market authorization holder's (MAH) global safety database aims to characterize the real-world safety profile of opicapone in elderly and very elderly PwP, thereby addressing an important evidence gap in this clinically relevant population.

## 2 Methods

This was a retrospective analysis of the Opicapone Global Safety Database using the validated Argus® platform, which enables systematic collection of post-authorization Individual Case Safety Reports (ICSRs) from multiple sources (e.g. healthcare professionals, PwP, and published literature) and automated reporting in compliance with ICH E2B standards. Cases were coded using MedDRA terminology. The database consolidates post-marketing AEs reported for opicapone since market introduction, providing a comprehensive longitudinal real-world dataset for descriptive safety analyses.

Data were retrieved from the Global Safety Database (data lock: 31 January 2026) and valid post-marketing ICSRs flagged as elderly, with opicapone as the suspected product, were included; clinical trial reports were excluded. Validity required an identifiable patient and reporter, a suspected product, and ≥ 1 AE. Cases were categorized as elderly (≥65–84 years) or very elderly (≥85 years), with age derived from exact values or assigned from decade midpoints. AEs were analyzed by MedDRA System Organ Class (SOC) and Preferred Term (PT), with seriousness classified in accordance with physician judgement. ‘Unlisted events’ were defined as those not included in section 4.8 of the European Opicapone Summary of Product Characteristics.

Analyses were descriptive and focused on patient demographics, overall reporting patterns, seriousness, the most frequently reported SOCs and PTs, and reports related to non-standard use (including off-label use, incorrect administration technique, as well as use with advanced infusion therapies), with comparisons between elderly and very elderly populations. Ethics committee approval and informed consent were not required, as this study used anonymized pharmacovigilance data.

## 3 Results

Of 4615 cases in the Global Safety Database, 3092 valid post-authorization ICSRs were flagged as involving elderly patients. Of these, 178 were excluded from age-stratified analyses because specific age was not reported, leaving 2914 cases (2558 elderly PwP and 356 very elderly PwP). Reports originated from multiple countries, with Japan representing the highest proportion of cases in both age groups, followed by the United States and several European countries (Table 1). Females accounted for 51% of elderly and 60% of very elderly; mean ages were 72.5 and 85.8 years, respectively.

**Table 1:** Demographic characteristics.

	Elderly ≥ 65–84 years (*n* = 2558)	Very Elderly ≥85 years (*n* = 356)
**Demographics**		
Age, mean, years	72.52	85.83
Sex Female, n (%) Male, n (%) Unknown, n (%)	1294 (51%) 1169 (46%) 95 (4%)	213 (60%) 122 (34%) 21 (6%)
**Geographic location**		
Japan, n (%)	1331 (52%)	268 (75%)
United States, n (%)	425 (17%)	44 (12%)
Spain, n (%)	282 (11%)	8 (2%)
Germany, n (%)	121 (5%)	10 (3%)
United Kingdom, n (%)	118 (5%)	14 (4%)
Italy, n (%)	98 (4%)	4 (1%)
Republic of Korea, n (%)	80 (3%)	5 (1%)
Portugal, n (%)	47 (2%)	3 (1%)
Other, n (%)	56 (2%)	0 (0%)

Across elderly and very elderly PwP (*n* = 2914), a total of 7195 AEs were reported, including 6511 events in elderly PwP and 684 events in very elderly PwP (Table 2). The majority of reported adverse events were non-serious, representing 81.4% (5301/6511) of events in elderly cases and 73.5% (503/684) of events in very elderly cases. At the SOC level, the most frequently reported categories in both age groups were “Nervous system disorders” and “Psychiatric disorders,” followed by “General disorders and administration-site conditions”, “Injury, poisoning and procedural complications,” and “Gastrointestinal disorders.” At the PT level, "Dyskinesia" and "Hallucination" were the most frequently reported AEs in both groups, with gastrointestinal events (e.g. nausea) and non-standard use related PTs more frequent in very elderly PwP. Hallucinations were the most frequently reported serious AEs in both groups (2.8–4.4%). Other reported serious events included dyskinesia and falls. Unlisted serious events (e.g. worsening of PD and delirium) were reported in less than 2%.

**Table 2:** Summary of reported adverse events and case management.

Adverse events	Elderly ≥ 65–84 years *N* = 6511 events	Very elderly ≥ 85 years *N* = 684 events
**Seriousness**		
Non-serious, % (n)	81.4% (5301)	73.5% (503)
Serious, % (n)	18.6% (1210)	26.5% (181)

**System organ class**		
Nervous system disorders, % (n)	26.4% (1720)	24.3% (166)
Psychiatric disorders, % (n)	17.6% (1147)	18.4% (126)
General disorders and administration site conditions, % (n)	15.3% (995)	9.9% (68)
Injury, poisoning and procedural complications, % (n)	9.8% (641)	15.5% (106)
Gastrointestinal disorders, % (n)	9.5% (616)	11.5% (79)

**Preferred term reported (top 10 in either group)**		
Dyskinesia, % (n)	7.1% (462)	7.7% (53)
Hallucination, % (n)	3.2% (211)	4.7% (32)
Hallucination, visual, % (n)	2.5% (163)	3.5% (24)
Therapeutic response shortened, % (n)	2.3% (148)	0.7% (5)
Constipation, % (n)	2.3% (148)	2.0% (14)
Dizziness, % (n)	2.2% (141)	2.2% (15)
Therapy cessation, % (n)	2.0% (133)	1.6% (11)
Nausea, % (n)	1.9% (124)	3.9% (27)
Tremor, % (n)	1.9% (121)	1.5% (10)
Non-standard use,^#^ % (n)	3.5% (225)	6.0% (41)
Somnolence, % (n)	1.7% (113)	3.2% (22)
Dysphagia, % (n)	1.4% (91)	2.8% (19)

**Serious preferred term reported (Top 10 in either group)**		
Hallucination, % (n)	2.8% (184)	4.4% (30)
Hallucination, visual, % (n)	2.27% (148)	3.5% (24)
Dystonia,^$^ % (n)	0.6% (38)	0.2% (1)
Dyskinesia, % (n)	0.52% (34)	0.4% (3)
Death, % (n)	0.5% (31)	0.9% (6)
Fall, % (n)	0.4% (26)	0.6% (4)
Delirium,^$^ % (n)	0.3% (17)	1.6% (11)
Worsening of Parkinson's disease,^$^ % (n)	0.3% (19)	0.9% (6)
Dementia,^$^ % (n)	0.4% (24)	0.2% (1)
Hallucination, auditory, % (n)	0.3% (19)	0.2% (1)
Pneumonia aspiration,^$^ % (n)	0.3% (16)	1.3% (9)
Hypotension, % (n)	0.05% (3)	0.7% (5)
Syncope, % (n)	0.2% (10)	0.7% (5)
Hospitalization, % (n)	0.2% (14)	0.6% (4)

**Action taken (case denominator)**	**Elderly** **≥** **65–84 years** **(*n*** **=** **2558)**	**Very Elderly** **≥85 years** **(*n*** **=** **356)**
Dose reduced, % (n)	1% (24)	2% (6)
Dose increased, % (n)	0% (5)	0% (0)
Dose not changed, % (n)	22% (574)	21% (75)
Drug withdrawn, % (n)	45% (1160)	49% (173)
Not applicable,* % (n)	4% (113)	6% (21)
Unknown, % (n)	27% (682)	23% (81)

Unless otherwise specified, percentages are calculated using the total number of reported events within each age group as the denominator. Percentages for treatment actions are based on the number of cases within each age group. ^#^Including use for a different indication (e.g., parkinsonism), incorrect posology, inappropriate administration mode (e.g., crushing tablets, open capsule), and concomitant use with infusion treatment options (LCIG and pLD/pCD); ^$^Unlisted events according to European or US Ongentys SmPC (EMA Ongentys, INN-opicapone SmPC; FDA Ongentys LABEL); *Cases for which no causal relationship has been established.

A total of 250 cases included reports of non-standard use. These comprised 225 events in the elderly group and 41 events in the very elderly group, representing 3.5% and 6.0% of all reported events, respectively. Reported issues included tablet crushing (*n* = 59) or capsule opening (*n* = 9), administration via PEG or nasogastric tube (*n* = 13), altered timing of administration (*n* = 132, including failure to maintain the recommended one-hour interval between opicapone and levodopa or taking the medication during the morning instead of at night), and altered daily dosage (*n* = 39). Off-label indications included parkinsonism (*n* = 40), essential tremor (*n* = 2), dementia with Lewy bodies (*n* = 1), and multiple system atrophy (*n* = 2). Use of opicapone in combination with infusion therapies was reported more frequently in elderly PwP than in very elderly PwP and included 63 cases with subcutaneous foslevodopa/foscarbidopa and 3 cases with levodopa-carbidopa intestinal gel. Among 66 infusion-related cases, 38 reported AEs, most commonly dyskinesia and hallucinations, followed by device-related infusion-site complications.

Treatment discontinuation was the most frequently reported action taken, occurring in 45% of elderly cases and 49% of very elderly cases. Dose reduction or increase was reported infrequently in both groups (≤2%). These patterns were similar between groups but information on event severity and the clinical rationale for these decisions was limited.

## 4 Discussion

This database analysis showed broadly similar reporting patterns of adverse events among elderly and very elderly PwP receiving opicapone, with no new safety signals identified. Most AEs were non-serious and aligned with the known opicapone profile [4]. The most frequently reported events were dopaminergic in nature or related to disease progression, and their distribution was broadly similar between the two age groups. This is consistent with increased susceptibility to dopaminergic adverse effects in older and frail PwP, particularly in the context of higher levodopa exposure (the pharmacological effect from COMT inhibition with opicapone).

Despite similarities in the overall spectrum of AEs, some differences in relative frequency were observed between age groups. At the PT level, shortening of the therapeutic response and constipation were more frequently reported in elderly PwP, whereas somnolence, and dysphagia were more common in very elderly PwP. Serious adverse events accounted for a greater proportion of reported events in very elderly than elderly PwP (26.5% vs 18.6% of events). Among these, hallucinations (including visual and auditory) were the most frequently reported, however, none occurred at a frequency greater than 5%. This may be partly explained by age-associated dopaminergic sensitivity, cognitive decline, and disease burden [5], rather than necessarily indicating a new opicapone-specific safety signal. Such effects may be more pronounced in individuals with age-related cognitive impairment or prior neuropsychiatric vulnerability.

Across both age groups, and despite the fact that most AEs were non-serious, management often involved treatment discontinuation, whereas dose modification was infrequent. This pattern suggests a more conservative approach in older PwP, favoring withdrawal over optimization (e.g. dose adjustment) and reflecting a ‘last in–first out’ strategy, although guidance generally recommends reducing dopamine agonists before altering levodopa-based regimens [6]. However, interpretation of this prescribing pattern is limited by the absence of AE severity data, which would typically guide decisions regarding treatment interruption, discontinuation, or dose adjustment. Non-standard use was more common in very elderly PwP and was primarily related to administration complexity, including tablet crushing (Japan only), capsule opening or dispersion, administration via PEG or nasogastric tube, and incorrect timing of dosing. Such variations may lead to faster absorption and altered pharmacokinetics without necessarily indicating inappropriate use; any impact may be mitigated by once-daily dosing and sustained COMT inhibition after treatment stabilization. Of note, the use of opicapone as an adjunct to levodopa infusion therapies was more frequently reported in the elderly group, where its use likely reflects attempts to improve the motor response, as infusion therapies often still require further optimization [7]. Device-aided therapies may, however, be less commonly used in very elderly or frail patients due to the burden of device management. In those PwP receiving concomitant infusion, AEs were mainly dopaminergic (dyskinesia and hallucinations) and similar to those seen with oral levodopa, except for device-related infusion-site complications.

Although a large proportion of reports originated from Japan, the type and distribution of AEs were consistent across geographical regions, with no evidence of region-specific safety signals. The higher number of reports from Japan likely reflects well-established differences in pharmacovigilance practices, including regulatory requirements for extensive post-marketing surveillance. This predominance of Japanese reports in the very elderly group may be considered both a strength, in terms of relevance to Japanese clinical practice, and a limitation, as country-specific reporting practices may influence age-group comparisons. Although PD is generally more prevalent in men worldwide (with reports of a female predominance in Japan [8]), women predominated in the very elderly group, in whom frailty, cognitive impairment, and treatment complexity are more common [2]. This predominance is likely driven by demographic factors and life-expectancy rather than differences in the opicapone safety profile. However, female sex has been associated with greater susceptibility to AEs including severe gastrointestinal dysfunction and a higher risk of clinically significant dysphagia, particularly at advanced age [9]. Consistent with this, a retrospective Italian study of opicapone reported a higher incidence of predominantly dopaminergic AEs in women, attributed to increased levodopa bioavailability [10]. Together, these factors complicate interpretation of the observed AE patterns in the very elderly population and make it difficult to disentangle the relative contributions of advanced age and sex.

Compared with spontaneous reporting systems primarily used for signal detection, this global MAH safety database enables a more comprehensive assessment of reporting patterns across age groups. Nevertheless, the analysis is subject to the inherent limitations of pharmacovigilance data, including under-reporting, reporting bias towards seriousness, variable data quality (limited clinical detail regarding disease severity, comorbidities and concomitant therapies) and the absence of a defined exposure denominator. Furthermore, the database does not contain age-specific exposure or person-time information. Consequently, reporting proportions cannot be interpreted as incidence rates or used to estimate the comparative risk of adverse events between age groups. Any differences should therefore be interpreted as descriptive reporting patterns rather than evidence of age-related differences in risk. As with all spontaneous pharmacovigilance analyses, individual case reports do not establish causality. Many reported events, including cognitive decline, delirium, falls, dysphagia and dementia, may reflect progression of advanced Parkinson's disease, comorbidities, frailty, or concomitant therapies rather than a direct effect of opicapone. In addition, the very elderly subgroup comprised 356 reported cases, and estimates derived from this subgroup may be subject to greater sampling variability than those from the larger elderly population. It is also acknowledged that differences in how opicapone is positioned within treatment algorithms across countries (e.g. if it is used as a later-line option in PwP who don't tolerate other generically available COMT inhibitors) may influence the tolerability profile observed in real-world reports. In contrast, clinical trials provide more controlled estimates of safety but in narrower patient populations.

Experience with other marketed peripheral inhibitors of COMT has shown a similar adverse effect profile, with some exceptions. For example, adjunctive entacapone can cause diarrhea as an idiosyncratic adverse reaction, reported to occur in 9% of patients treated for up to 3 years [11]. In the post-marketing experience of tolcapone, four cases of fulminant liver failure were encountered, leading to strict regulation of this COMT inhibitor [12]. Neither of these problems have been observed in our pharmacovigilance analysis of opicapone. In conclusion, this analysis found broadly similar adverse-event reporting patterns in elderly and very elderly PwP receiving opicapone in routine clinical practice. Most reported AEs were non-serious and consistent with the established safety profile of opicapone, supporting continued use in older populations with appropriate clinical monitoring.

## Study funding

This study was funded by BIAL - Portela & Ca, S.A.

## Data sharing statement

All available data are contained within this short report.

## CRediT authorship contribution statement

**Taku Hatano:** Writing – review & editing. **Peter A. LeWitt:** Writing – review & editing. **Tetsuya Maeda:** Writing – review & editing. **Javier Pagonabarraga Mora:** Writing – review & editing. **Bruno F. Dias:** Writing – review & editing, Formal analysis. **Helena C. Brigas:** Writing – original draft, Methodology, Formal analysis. **Diogo Lopes:** Writing – review & editing, Methodology. **Helena Gama:** Writing – review & editing. **Joerg Holenz:** Writing – review & editing, Supervision.

## Ethics declaration

The authors have NOT obtained informed consent from participants or their legal representatives. Informed consent was not required because this study was based on the secondary analysis of anonymized individual case safety reports collected through routine pharmacovigilance activities.

This study was performed in compliance with relevant laws, regulatory frameworks and guidelines where the research took place.

Data were obtained from a global safety database containing anonymized post-marketing individual case safety reports. Ethics committee approval and informed consent were not required because the study involved the secondary analysis of anonymized pharmacovigilance data and did not include direct participant involvement or identifiable patient information.

## Declaration of competing interest

Taku Hatano reports research funding from the Japan Agency for Medical Research and Development; consultancy fees from Sumitomo Pharma, Orion Pharma, Ajinomoto Co., and Lundbeck Japan K.K.; and honoraria for speaking from Kyowa Kirin Co., Ltd., Takeda Pharmaceutical Co., Ltd., Eisai Co., Ltd., FP Pharmaceutical Co., Ltd., Sumitomo Pharma Co., Ltd., Argenx Japan, Merck & Co., Inc., Novartis Pharma K.K., AbbVie Inc., Ono Pharmaceutical Co., Ltd., EA Pharma Co., Ltd., and Nihon Medi-Physics Co., Ltd. He is a member of the Parkinsonism and Related Disorders editorial board. Peter A. LeWitt has served as a compensated consultant to Neurocrine Biosciences, Inc., and as a Steering Committee member for Neurocrine's OPTI-ON clinical trial. Tetsuya Maeda has served as a medical expert in the Japanese phase III clinical development program of opicapone and reports no other conflicts of interest related to the present work. Javier Pagonabarraga Mora reports no conflicts of interest. Bruno F. Dias, Helena C. Brigas, Diogo Lopes, Helena Gama, and Joerg Holenz are employees of BIAL.
